# Epidemiology and Clinical Course of First Wave Coronavirus Disease Cases, Faroe Islands

**DOI:** 10.3201/eid2703.202589

**Published:** 2021-03

**Authors:** Marnar F. Kristiansen, Bodil H. Heimustovu, Sanna á Borg, Tróndur Høgnason Mohr, Hannes Gislason, Lars Fodgaard Møller, Debes H. Christiansen, Bjarni á Steig, Maria Skaalum Petersen, Marin Strøm, Shahin Gaini

**Affiliations:** National Hospital of the Faroe Islands, Tórshavn, Faroe Islands (M.F. Kristiansen, B.H. Heimustovu, S. á Borg, T.H. Mohr, B. á Steig, S. Gaini);; Ministry of Health COVID-19 Task Force, Tórshavn (M.F. Kristiansen, B.H. Heimustovu, B. á Steig);; University of the Faroe Islands, Tórshavn (M.F. Kristiansen, H. Gislason, M.S. Petersen, M. Strøm, S. Gaini);; Office of the Chief Medical Officer, Tórshavn (L.F. Møller);; Faroese Food and Veterinary Authority, Tórshavn (D.H. Christiansen);; The Faroese Hospital System, Tórshavn (M.S. Petersen);; Statens Serum Institut, Copenhagen, Denmark (M. Strøm);; Odense University Hospital, Odense, Denmark (S. Gaini);; University of Southern Denmark, Odense (S. Gaini)

**Keywords:** Contact tracing, quarantine, clinical characteristics, epidemiology, superspreader events, elimination, reproduction number, serial interval, surveillance, respiratory infections, severe acute respiratory syndrome coronavirus 2, SARS-CoV-2, SARS, COVID-19, coronavirus disease, zoonoses, viruses, coronavirus, Faroe Islands

## Abstract

The Faroe Islands was one of the first countries in the Western Hemisphere to eliminate coronavirus disease (COVID-19). During the first epidemic wave in the country, 187 cases were reported between March 3 and April 22, 2020. Large-scale testing and thorough contact tracing were implemented early on, along with lockdown measures. Transmission chains were mapped through patient history and knowledge of contact with prior cases. The most common reported COVID-19 symptoms were fever, headache, and cough, but 11.2% of cases were asymptomatic. Among 187 cases, 8 patients were admitted to hospitals but none were admitted to intensive care units and no deaths occurred. Superspreading was evident during the epidemic because most secondary cases were attributed to just 3 infectors. Even with the high incidence rate in early March, the Faroe Islands successfully eliminated the first wave of COVID-19 through the early use of contact tracing, quarantine, social distancing, and large-scale testing.

The World Health Organization declared coronavirus disease (COVID-19) a pandemic on March 12, 2020 (*1*). Initial outbreaks were reported in China during late 2019, and by February 2020 COVID-19 had spread globally and caused clusters of contagion in Europe (*2*).

The first confirmed case of COVID-19 in the Faroe Islands was identified on March 3. The Faroe Islands, located in the North Atlantic Ocean, is a high-income self-governing country in the Kingdom of Denmark with a population of 52,428 (*3*). During March 3–April 22, 2020, 187 persons in the Faroe Islands tested positive for COVID-19 (Figure 1). The last case was diagnosed on April 22 and recovered on May 8, at which point the first wave of COVID-19 ended in the country. To eliminate COVID-19, the Faroe Islands used an active suppression strategy that included large-scale testing, contact tracing, quarantine, and social distancing measures.

**Figure 1:**
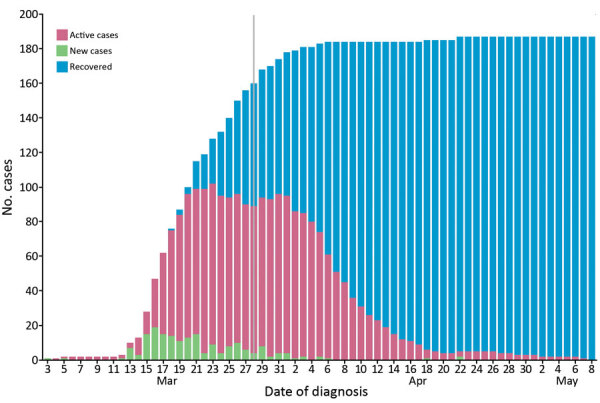
All confirmed cases of coronavirus disease in the Faroe Islands as of May 8, 2020. Active cases, recovered cases, new cases per day, and cumulative cases are shown. Vertical gray line indicates change in recovery criteria on March 28, which prolonged the required time for recovery to >14 days.

We describe the epidemiology and clinical course of COVID-19 during March 3–May 8, 2020, and the successful elimination of the first wave of COVID-19 in the Faroe Islands. We assessed the effects of contact tracing, quarantine, and social distancing. We also estimated the average and observed number of secondary cases caused by each infector at the date of diagnosis during various stages of the epidemic.

## Methods

### Identification of COVID-19 Cases and Contacts

**Figure 2:**
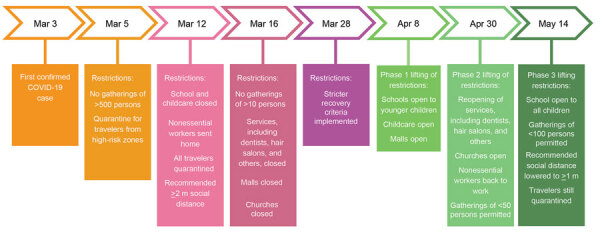
Timeline of government actions taken against COVID-19, Faroe Islands. Restrictions were not mandatory but generally were followed by the public. It is difficult to conclude which effect every specific nonpharmaceutical intervention had on the Faroese epidemic as several were implemented successively and some in parallel, although these interventions in concordance with contact tracing and quarantine managed to eliminate the first wave of the epidemic. COVID-19, coronavirus disease.

The government of Faroe Islands implemented lockdown on March 12, 2020, when only 3 confirmed cases were known in the country. The main nonpharmaceutical interventions were closing schools, childcare centers, and nonessential public workplaces. The government discouraged unnecessary travel and reduced transport to and from the country to a minimum. The government also promoted social distancing, frequent handwashing and use of hand sanitizers, and avoiding large gatherings. After March 12, all travelers arriving in the Faroe Islands were asked to self-quarantine for 14 days (Figure 2). Government authorities implemented all measures as nonmandatory recommendations, which the public generally followed.

The Faroe Islands quickly adapted diagnostic real-time reverse transcription PCR (RT-PCR) resources to test for severe acute respiratory syndrome coronavirus 2 (SARS-CoV-2), the virus that causes COVID-19. RT-PCR resources normally used in salmon farming by the Food and Veterinary Authority were adapted to implement a large-scale COVID-19 testing strategy early in the epidemic. This strategy enabled high testing capacity per capita; 600 tests per day were administered during the first days of the outbreak, and test results were available within 1–2 days.

The Office of the Chief Medical Officer performed contact tracing by requesting that all persons with positive RT-PCR test results self-isolate and list persons with whom they had close contact <48 hours before symptom onset. Asymptomatic positive persons were asked to list all contacts <48 hours before diagnosis. For contact tracing, close contacts were persons who had face-to-face contact <2 meters of a positive case for >15 minutes; direct physical contact with a case; direct care of a COVID-19 patient without using proper personal protective equipment; or other equally assessed exposures, such as living in a household with, having face-to-face contact for >15 minutes with, or riding in a vehicle with a confirmed COVID-19 case-patient (*4*). The Office of the Chief Medical Officer contacted all reported close contacts and requested that they quarantine for 14 days. If persons could not quarantine at home, the government offered hotel rooms free of charge to both cases and contacts.

The Ministry of Health established a COVID-19 task force (CTF) of medical doctors to maintain contact with all isolated COVID-19 cases and quarantined contacts. To monitor for symptom development and clinically evaluate whether cases needed to be hospitalized, task force members contacted diagnosed cases at intervals of <48 hours during isolation until the end of the quarantine period. CTF recorded information on the infection source, including whether the case was contracted from a known infector, an imported case, or an unknown source. CTF also recorded information on quarantine before RT-PCR testing and the number of close contacts asked to quarantine. CTF recorded symptoms for all patients prospectively. Reported symptoms included cough, headache, throat pain, dyspnea, and fever. CTF also recorded the dates of illness onset, end of acute symptoms, and end of quarantine for patients.

Quarantined contacts were given a telephone number to call if symptoms developed. Shortly before the end of their quarantine, contacts were asked again whether symptoms had developed to determine whether they should be tested. Some asymptomatic contacts also were tested, but this was not done routinely. Testing required a referral from a doctor.

During the initial epidemic period, recovery criteria in the Faroe Islands followed guidelines from Denmark and considered persons who were without symptoms for >48 hours recovered. However, because observations indicated that 48 hours without symptoms did not ensure that the infectious period was over, recovery criteria were changed on March 28 to >14 days after a positive RT-PCR. Retesting was not recommended for positive cases and negative tests were not used as part of the recovery criteria.

### Statistical Analyses

The serial interval is the time from symptom onset in a primary case to symptom onset in a secondary case. The generation time is the time between infection events in a primary case and a secondary case. Generation time is difficult to observe but is expected to be approximately equal to the serial interval (*5*,*6*). We chose to use the serial interval in all cases in this study and we estimated the mean serial interval by using the EpiEstim package in R (R Foundation for Statistical Computing, https://www.r-project.org). We assumed a gamma distributed model on 124 identified infector–infectee pairs for which symptom onset was known for both cases.

The reproduction number (R_0_) is the average number of secondary cases each case will infect. A time-varying reproduction number (R_t_) is the average number of secondary cases caused by each primary case at different times in the epidemic. R_t_ can be affected by government interventions, behavior changes, or when a certain fraction of the population is no longer susceptible to the pathogen because of immunity. We estimated R_t_ by using sliding 1-week windows, which assumes the transmission potential at given time t is the same as in the time window that ends at time t. We used the default 1-week window of the EpiEstim package (*7*) and took the average of the transmission potential of that sliding window to estimate R_t_. Using sliding windows reduces noise while retaining the possibility to show changes in real-time in different phases in the epidemic. We used local serial interval data and the distribution of local and imported case counts as input data.

We determined the observed individual reproductive number (R_obs_) by using transmission chains in the Faroe Islands. R_obs_ is the average number of observed secondary infections caused by each primary case at different times in the epidemic by date of diagnosis.

We made 2 adjustments make R_t_ and R_obs_ data comparable. For cases of unknown transmission, we set the infector as diagnosed 5 days earlier by rounding the serial interval from 5.35 to 5 days to avoid underestimating R_obs_ by censoring these cases. Because R_obs_ is based on the infector and R_t_ is based on the infectee, we displaced R_obs_ forward by the serial interval of 5 days to facilitate visual comparison of R_obs_ and R_t_.

### Determining Transmission Chains

We determined transmission chains by interviewing newly diagnosed patients about their contacts and whereabouts 2 weeks before symptom onset and linking this with information on previously known cases. If multiple exposures were known for a case and the most likely infector was uncertain, we chose the earliest diagnosed case as infector. Persons who had been abroad during the previous 14 days were classified as imported cases if no better explanation was known. When cases could not be linked to previous cases and had no recent travel history, we classified the transmission as unknown.

All study procedures were in accordance with the Declaration of Helsinki. The study was approved by the Data Protection Authority of the Faroe Islands (approval no. 20/00096-12).

## Results

In the first wave of COVID-19, 187 cases were identified; the first case on March 3 and the last on April 22. On May 8, the Faroe Islands had no active COVID-19 cases. No fatalities or admissions to the intensive care unit occurred during this first wave. By May 8, a total of 7,653 RT-PCR tests for SARS-CoV-2 had been performed on 6,957 persons and the Faroe Islands had a per capita testing rate of 13,339/100,000 population, the highest globally (*8*,*9*). Furthermore, at that time, the Faroe Islands had the 12th highest confirmed cases per capital 357/100,000 population (*8*,*9*) (Table).

**Table:** Occurrence, characteristics, and symptoms of 187 coronavirus disease cases during March 3–May 8, 2020, Faroe Islands*

Variable	COVID-19 cases						
	All cases	Sex			Age range, y		
		Male	Female		0–17	18–64	>65
All cases	187	88 (47.1)	99 (52.9)		24 (12.8)	143 (76.5)	20 (10.7)
Cases/100,000 population	357	324	393		184	478	218
Cases tested by RT-PCR	6,957	3,091 (45.5)	3,866 (54.5)		1,132 (19.3)	4,965 (68.4)	860 (12.3)
RT-PCR tests/100,000 population	13,339	11,377	15,305		8,660	16,597	9,381
Reported symptoms							
Asymptomatic	21 (11.2)	11 (12.5)	10 (10.1)		6 (25.0)	9 (6.3)	6 (30.0)
Fever	118 (63.1)	55 (62.5)	63 (63.6)		12 (50.0)	94 (65.7)	12 (60.0)
Cough	83 (44.4)	47 (53.4)	36 (36.4)		4 (16.7)	71 (49.7)	8 (40.0)
Headache	89 (47.6)	36 (40.9)	53 (53.5)		5 (20.8)	78 (54.5)	6 (30.0)
Sore throat	56 (29.9)	24 (27.3)	32 (32.3)		3 (12.5)	51 (35,7)	2 (10.0)
Dyspnea	20 (10.7)	7 (8.0)	13 (13.1)		1 (4.2)	19 (13.3)	0 (0.0)
Loss of smell or taste†	63 (33.7)	23 (26.1)	40 (40.4)		3 (12.5)	56 (39.2)	4 (20.0)
Fatigue†	26 (13.9)	11 (12.5)	15 (15.2)		0 (0.0)	23 (16.1)	3 (15.0)
Rhinorrhea†	44 (23.5)	21 (23.9)	23 (23.2)		4 (16.7)	35 (24.5)	5 (25.0)
Body aches†	36 (19.3)	22 (25.0)	14 (14.1)		1 (4.2)	30 (21)	5 (25.0)
Chest tightness†	15 (8.0)	8 (9.1)	7 (7.1)		1 (4.2)	14 (9.8)	0 (0.0)
Diarrhea†	11 (5.9)	2 (2.3)	9 (9.1)		2 (8.3)	6 (4.2)	3 (15.0)
Abdominal pain†	11 (5.9)	4 (4.5)	7 (7.1)		3 (12.5)	4 (2.8)	4 (20.0)

*Values are no. (%) except as indicated. COVID-19, coronavirus disease; RT-PCR, reverse transcription PCR. †Only recorded when mentioned by patients. Other symptoms were systematically collected.

Among identified case-patients, 88.8% experienced symptoms, the most prevalent of which were fever (63.1%), headache (47.6%), and cough (44.4%) (Table). More asymptomatic cases occurred among persons <18 (25%) and >65 years of age (30%) than among persons 18–64 years of age (6.3%) (Table). The mean time from symptom onset to diagnosis was 3.06 days (range <1–17 days; 95% CI 2.67–3.45 days); 6 cases were diagnosed before the onset of symptoms. The median age among case-patients was 40 years (range 0–92 years) (Table).

Among 187 cases, 8 patients were hospitalized, and the median length of hospitalization was 2 days (range 0–11 days). The median age of hospitalized case-patients was 57 years (range 37–92 years); and 1 patient was hospitalized twice. Among the 8 hospitalized case-patients, 7 had >1 underlying condition, including hypertension, emphysema, asthma, ulcerative colitis, diabetes, and cardiovascular disease.

**Figure 3:**
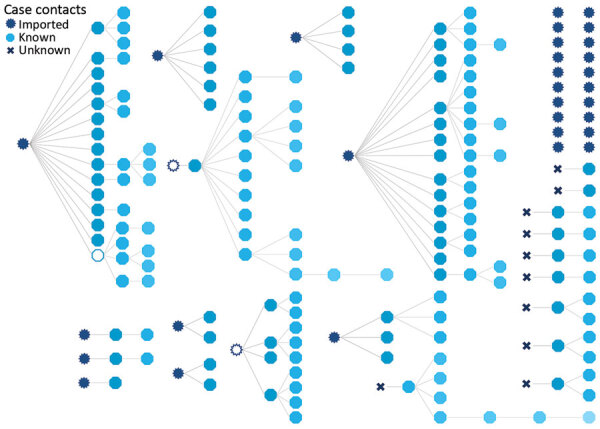
Transmission chains of coronavirus disease, Faroe Islands. All transmission chains are shown but are not represented chronologically. Transmission is based on persons, not events. The 3 open symbols represent known cases that were not tested or that tested negative for coronavirus. Blue shading in hectogons denotes secondary, tertiary, and quaternary cases infected from primary case. When multiple exposures were known for a case, the first exposure was chosen as the source of infection; this choice might slightly overestimate the number of secondary cases caused some infectors. The 20 cases shown in the top right were imported and led to no further infections. Among 9 cases that originated from contact with known but untested persons or persons with negative test results (denoted by open circles), we presume the tests were false negative; those who were not tested had relevant symptoms and contact with later cases but had left the country or the course of the disease was over before their case was discovered. We classified cases that caused >10 secondary infections superspreading cases.

We noted 10 cases of unknown or uncertain origin and 9 from known contact with a person who was not tested or who tested negative (Figure 3). We classified 30 cases as imported; 62 cases were acquired in a household, 39 in a workplace, 11 during an event, and 45 in other or unknown settings. Among imported cases, 20 did not cause further infections. We noted 4 large transmission chains that led to 105 other cases. We also noted 3 superspreading cases, each of which infected >10 secondary cases.

**Figure 4:**
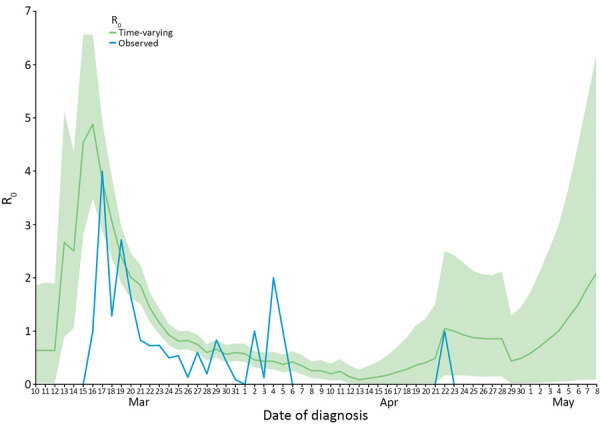
Time-varying reproduction number (R_t_) and observed reproduction number (R_obs_) for coronavirus disease by date, Faroe Islands. Green shading indicates 95% CI for R_t_. We noted a rapid decrease in R_t_, from 4.88 on March 16. From March 24 onward, R_t_ and R_obs_ were <1 until April 22 when the last case was confirmed in the Faroe Islands. After May 4, R_t_ rose >1 due to increasing uncertainty in the estimate. We calculated R_t_ by using the EpiEstim package in R (R Foundation for Statistical Computing, https://www.r-project.org) and local data on serial interval and imported cases. R_obs_ was determined by information from the transmission chains. We made 2 adjustments to compare R_obs_ to R_t_: we moved R_obs_ 5 days forward (equal to the serial interval) because R_obs_ is measured on the infector; and we set R_t_ on the infected case. When the infector was unknown, we set transmission as 5 days earlier, equal to the serial interval, to avoid underestimating R_obs_ by censoring those cases. R_0_, reproduction number; R_obs_, observed reproduction number; R_t_, time-varying reproduction number.

We estimated the serial interval by fitting a gamma distribution on symptom onset of infector–infectee pairs, resulting in a mean of 5.36 days (95% CI 4.63–6.09 days; SD 4.12 days, 95% CI 3.56–4.93 days). R_t_ peaked at 4.88 on March 16, after which it fell to <1 from March 24 onward. On April 22, we saw a short peak of >1 with the last case. After the last case, R_t_ rose to >1 again on May 4, even though no new cases were detected, but the 95% CI was quite large (95% CI 0.06–2.99). R_obs_ roughly followed R_t_ when displaced by the serial interval with a peak of 4.0 on March 17 (Figure 4).

**Figure 5:**
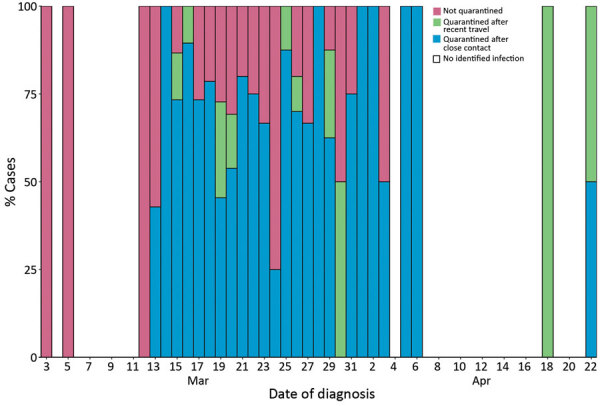
Percentage of known coronavirus disease cases quarantined by date, Faroe Islands. During March 3–12, 2020, no cases were quarantined because no previous infection was diagnosed in the Faroe Islands and travel quarantine was not enforced yet. After March 12, most cases were quarantined, either as a result of recent travel or close contact with a positive case. However, some nonquarantined cases persisted and an unquarantined case was identified on April 3.

**Figure 6:**
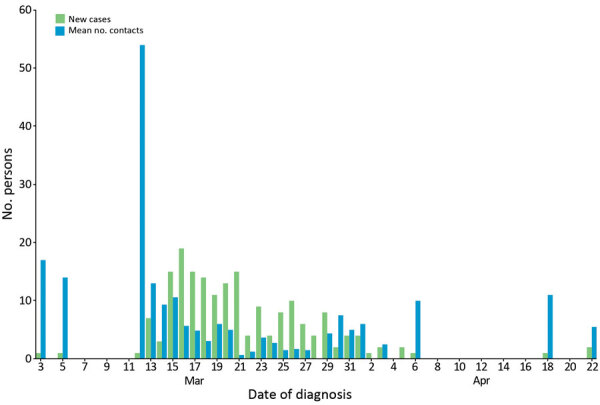
Mean number of contacts per coronavirus disease case placed in quarantine each day, Faroe Islands. The number of close contacts per case quickly dropped after March 12, 2020, and the effects of social distancing due to government measures, changes in social behaviors, and quarantine is apparent.

During March 2–April 22, a total of 854 persons were quarantined because of close contact with a COVID-19 case; 132 (15%) were later confirmed as having COVID-19 cases. Fourteen persons were quarantined before diagnosis because of recent travel (Figure 5). For each identified case, the mean number of contacts quarantined was 5.1 (Figure 6).

## Discussion

The Faroe Islands were one of the first countries in the Western Hemisphere to eliminate COVID-19, showing the feasibility of elimination in a country with well-defined borders, even starting with a high incidence. Testing, contact tracing, quarantine, and social distancing measures were instrumental to success in the Faroe Islands. These strategies have proven effective in suppressing the infection in other countries, including Iceland, Taiwan, Switzerland, and New Zealand (*10*–*13*). A notable success is that only 10.7% of COVID-19 cases in the Faroe Islands were among persons >65 years of age, even though this group constitutes 17.6% of the population (*3*). Low incidence among persons >65 years of age reflects the timely government restrictions on access to care homes, nursing homes, and hospitals, which might explain why no COVID-19 deaths or intensive care unit admissions occurred and only 8 case-patients were admitted to hospitals during the first wave in the Faroe Islands.

After the initial success of eliminating COVID-19, government travel restrictions remained strict, and a 14-day quarantine was recommended for travelers arriving in the country. Travel restrictions were loosened on June 15, quarantine was no longer requested, and only 1 test was required at the border. Lifting travel restrictions did not lead to an instant influx of cases, but some sporadic cases were found among tourists at the borders and foreign workers at harbors in the Faroe Islands. However, on August 4, two locally transmitted cases of unknown origin put an end to a streak of 104 days without locally transmitted cases.

The number of close contacts put in quarantine fell quickly after government recommendations were implemented (Figure 6). After the outbreak’s initial days, implemented restrictions resulted in quarantine of most new cases before diagnosis because of travel or contact with a previous case (Figure 5). This finding demonstrates that contact tracing and quarantine were effective strategies, despite some cases persisting without quarantine. Unquarantined cases were among cases of unknown origin or contacts not included in the close contact quarantine guidelines. Cases diagnosed outside of quarantine might indicate that contact tracing and quarantine would not have been enough to eliminate the epidemic without simultaneously implementing social distancing measures.

Mapping the transmission chains of COVID-19 revealed that most cases infected few or no secondary contacts, whereas 3 superspreading cases set off long, aggressive chains that led to most of the identified secondary locally transmitted cases. When we mapped transmission chains, among cases that had multiple exposures but the most likely infector was unclear, we chose the first diagnosed case in the chain as the infector, which might slightly overestimate the number of secondary cases caused by some infectors.

The observation of superspreading persons aligns with previous findings in many infectious disease outbreaks, including the 2002–2003 SARS outbreaks, in which a small percentage of cases in a population caused most transmission events, known as the 20/80 rule (*14*). Our observations support other reports that indicated super-spreading has played a major role in the current outbreak of COVID-19 (*15*).

Variation in demonstrated infectiousness can be affected by host, pathogen, or the environment. The 3 superspreading cases in our study had many sporadic contacts, were of varying ages and of both sexes, and had no underlying conditions. Although we do not have data to speculate on why these persons spread the disease more effectively than others, known risk factors for superspreading described in the literature for other infectious disease epidemics include co-infections, a higher viral load in superspreaders, or that superspreaders had more close contacts than other cases (*14*,*16*). Other hypotheses for these apparent differences in COVID-19 spread could be that some transmission chains in the Faroe Islands had more contagious strains of SARS-CoV-2 than others, which other preliminary studies might support (*17*,*18*). Further studies, including sequencing of SARS-CoV-2 viruses from the Faroe Islands, will further investigate these aspects.

Of note, infection in the Faroe Islands appears to have been spread by a small number of quarantined children who tested negative, presumably because of false-negative tests. The children were exposed independently and were quarantined with their family members who later tested positive for SARS-CoV-2 without exposure to positive cases themselves. The children continued to test negative with repeated tests.

The Faroe Islands are a unique place to investigate the effects of COVID-19. Because of large-scale testing in the country, few unrecorded cases would be expected, and this was confirmed by seroprevalence study. The study, conducted during April–May in a representative 2% sample of the population, assessed SARS-CoV-2 seroprevalence at 0.7%, indicating only a few unrecorded cases (*19*). The performance and sensitivity of RT-PCR tests in community settings has been in doubt because the likelihood of a false-negative test is assumed to be higher among persons with mild or no symptoms compared with hospitalized patients. However, our practical experience shows that elimination is possible with large-scale testing, even if some cases might be missed due to false-negative results. One consequence of the intensive testing regime in the Faroe Islands is that clinical data reflect symptoms in the milder spectrum of COVID-19 disease. Studies in other countries might overestimate the prevalence of severe symptoms because severe cases are more likely to be tested in those settings, and some milder cases might be missed.

Studies from other countries have shown proportions of asymptomatic cases ranging from 11.9% to 51.7% (*12*,*20*–*22*). We found 11.2% of cases in our study were asymptomatic. One reason for the difference might be that COVID-19 symptoms initially were used as criteria for testing in the Faroe Islands and some asymptomatic cases might have been missed. Another explanation of the different proportion of asymptomatic cases might be misclassification of symptoms in previous reports from the country, meaning COVID-19 cases categorized as asymptomatic patients were presymptomatic. The most prevalent symptoms reported by COVID-19 cases in our study were fever, headache, and cough, similar to findings in other studies (*12*,*21*,*23*,*24*).

In the Faroe Islands, both R_t_ and R_obs_ showed a rapid decrease as effects of social distancing, contact tracing, and quarantine were established, which indicates that the measures had the desired effect. Toward the end of the epidemic, after May 4, R_t_ increased to >1 and had an increasingly high 95% CI, even though no new cases were detected after April 22. R_t_ should not increase without new cases, but the increase seen here is likely due to the small size of the dataset and increasing uncertainty.

If R_0_ falls to <1, an epidemic will die out, indicating that measures to suppress the spread are working. Changes in R_0_ should be interpreted with caution, and assigning causal effects to specific government measures is challenging because several measures were implemented at the same time or over short periods (Figure 2); their effects on the contagion only can be seen after some delay. The changes in individual behavior caused by the media focus on the pandemic probably also have had an independent effect from any government measures. Furthermore, the statistical methods we used frequently overestimate R_0_ in the early stages of an epidemic, which would make the decrease in R_0_ seem more rapid than it was (*25*).

Most countries have pursued a strategy to mitigate the spread of COVID-19 and flatten the epidemic curve, but others, such as New Zealand, announced a goal to eliminate COVID-19 (*13*,*26*). The Faroe Islands successfully eliminated COVID-19 on May 8, 2020, but because controls on incoming travelers were reduced, elimination did not last (*27*).

A strength of this study is the use of nationwide data that includes all confirmed cases and prospective reporting of symptoms, which gives a more accurate description of COVID-19 symptoms compared with studies focusing on admitted patients. Furthermore, because the Faroe Islands had some of the world’s highest per capita testing rates, few unreported cases could be expected, strengthening the representation of the general course of the illness in the country.

Limitations of our study include the limited sensitivity of oropharyngeal swabs used for RT-PCR, which might lead to false-negative test results and, thus, underestimating the total number of cases. However, a follow-on seroprevalence study in the Faroe Islands indicated few unrecorded infections (*19*). With 187 cases, no fatalities, and few hospital admissions, ascertaining much about severe COVID-19 in the Faroe Islands is difficult, but the country shows a good representation of the most general course of disease. The Faroe Islands only have sea borders, and COVID-19 elimination here might not be readily generalizable to countries with land borders because control of incoming travelers can be more difficult in such settings.

In conclusion, our study includes all nationwide cases during the first wave of COVID-19 in the Faroe Islands, adds to the knowledge of COVID-19 symptoms in mild cases, and further supports to the role of superspreading in the pandemic. An effective suppression strategy led to eliminating the first wave of COVID-19 in the Faroe Islands, but the infection reappeared after the borders were reopened. This reemergence is indicative that other countries with easily monitored borders could feasibly eliminate COVID-19 by using a combination of large-scale testing, contact tracing, social distancing measures, and border restrictions. The rise of a second COVID-19 wave also is a warning that relaxing border restrictions will lead to a rise in infections.

## References

[1] World Health Organization. WHO Director-General’s opening remarks at the media briefing on COVID-19—11 Mar 2020 [cited 2021 Jan 12]. https://www.who.int/director-general/speeches/detail/who-director-general-s-opening-remarks-at-the-media-briefing-on-covid-19---11-march-2020

[2] Spiteri G, Fielding J, Diercke M, Campese C, Enouf V, Gaymard A, et al. First cases of coronavirus disease 2019 (COVID-19) in the WHO European Region, 24 January to 21 February 2020. Euro Surveill. 2020;25:2000178. 10.2807/1560-7917.ES.2020.25.9.2000178 32156327 PMC7068164

[3] Hagstova Føroya. Population statistics, Faroe Islands [cited 2021 Jan 12]. https://hagstova.fo/en/population/population/population-0

[4] World Health Organization. Coronavirus disease 2019 (COVID-19) situation report–61; 2020 Mar 21 [cited 2021 Jan 12]. https://apps.who.int/iris/handle/10665/331605

[5] Griffin J, Casey M, Collins Á, Hunt K, McEvoy D, Byrne A, et al. Rapid review of available evidence on the serial interval and generation time of COVID-19. BMJ Open. 2020;10:e040263. 10.1136/bmjopen-2020-040263 33234640 PMC7684810

[6] Ma S, Zhang J, Zeng M, Yun Q, Guo W, Zheng Y, et al. Epidemiological parameters of COVID-19: case series study. J Med Internet Res. 2020;22:e19994. 10.2196/19994 33001833 PMC7553786

[7] Thompson RN, Stockwin JE, van Gaalen RD, Polonsky JA, Kamvar ZN, Demarsh PA, et al. Improved inference of time-varying reproduction numbers during infectious disease outbreaks. Epidemics. 2019;29:100356. 10.1016/j.epidem.2019.100356 31624039 PMC7105007

[8] Roser M, Ritchie H, Ortiz-Ospina E, Hasell J. Coronavirus pandemic (COVID-19). Our World in Data [cited 2021 Jan 12]. https://ourworldindata.org/covid-cases

[9] Coronavirus in the Faroe Islands—statistics [in Danish] [cited 2021 Jan 12]. https://corona.fo/statistics

[10] Salathé M, Althaus CL, Neher R, Stringhini S, Hodcroft E, Fellay J, et al. COVID-19 epidemic in Switzerland: on the importance of testing, contact tracing and isolation. Swiss Med Wkly. 2020;150:w20225. 10.4414/smw.2020.20225 32191813

[11] Cheng H-Y, Jian S-W, Liu D-P, Ng T-C, Huang W-T, Lin H-H; Taiwan COVID-19 Outbreak Investigation Team. Contact tracing assessment of COVID-19 transmission dynamics in Taiwan and risk at different exposure periods before and after symptom onset. JAMA Intern Med. 2020;180:1156–63. 10.1001/jamainternmed.2020.2020 32356867 PMC7195694

[12] Gudbjartsson DF, Helgason A, Jonsson H, Magnusson OT, Melsted P, Norddahl GL, et al. Spread of SARS-CoV-2 in the Icelandic population. N Engl J Med. 2020;382:2302–15. 10.1056/NEJMoa2006100 32289214 PMC7175425

[13] Baker MG, Wilson N, Anglemyer A. Successful elimination of Covid-19 transmission in New Zealand. N Engl J Med. 2020;383:e56. 10.1056/NEJMc2025203 32767891 PMC7449141

[14] Stein RA. Super-spreaders in infectious diseases. Int J Infect Dis. 2011;15:e510–3. 10.1016/j.ijid.2010.06.020 21737332 PMC7110524

[15] Gómez-Carballa A, Bello X, Pardo-Seco J, Martinón-Torres F, Salas A. Mapping genome variation of SARS-CoV-2 worldwide highlights the impact of COVID-19 super-spreaders. Genome Res. 2020;30:1434–48. 10.1101/gr.266221.120 32878977 PMC7605265

[16] Shen Z, Ning F, Zhou W, He X, Lin C, Chin DP, et al. Superspreading SARS events, Beijing, 2003. Emerg Infect Dis. 2004;10:256–60. 10.3201/eid1002.030732 15030693 PMC3322930

[17] Tang X, Wu C, Li X, Song Y, Yao X, Wu X, et al. On the origin and continuing evolution of SARS-CoV-2. Natl Sci Rev. 2020 Mar 3;nwaa036. 10.1093/nsr/nwaa036 PMC7107875 34676127

[18] Koyama T, Platt D, Parida L. Variant analysis of COVID-19 genomes. Bull World Health Organ. 2020 Feb 24 [Epub ahead of print]. 10.2471/BLT.20.253591 PMC7375210 32742035

[19] Petersen MS, Strøm M, Christiansen DH, Fjallsbak JP, Eliasen EH, Johansen M, et al. Seroprevalence of SARS-CoV-2–specific antibodies, Faroe Islands. Emerg Infect Dis. 2020;26:2761–3. 10.3201/eid2611.202736 32726200 PMC7588539

[20] Mizumoto K, Kagaya K, Zarebski A, Chowell G. Estimating the asymptomatic proportion of coronavirus disease 2019 (COVID-19) cases on board the Diamond Princess cruise ship, Yokohama, Japan, 2020. Euro Surveill. 2020;25:2000180. 10.2807/1560-7917.ES.2020.25.10.2000180 32183930 PMC7078829

[21] Zhu J, Ji P, Pang J, Zhong Z, Li H, He C, et al. Clinical characteristics of 3062 COVID-19 patients: A meta-analysis. J Med Virol. 2020;92:1902–14. 10.1002/jmv.25884 32293716 PMC7262119

[22] Lavezzo E, Franchin E, Ciavarella C, Cuomo-Dannenburg G, Barzon L, Del Vecchio C, et al.; Imperial College COVID-19 Response Team; Imperial College COVID-19 Response Team. Suppression of a SARS-CoV-2 outbreak in the Italian municipality of Vo’. Nature. 2020;584:425–9. 10.1038/s41586-020-2488-1 32604404 PMC7618354

[23] Guan WJ, Ni ZY, Hu Y, Liang WH, Ou CQ, He JX, et al.; China Medical Treatment Expert Group for Covid-19. Clinical Characteristics of Coronavirus Disease 2019 in China. N Engl J Med. 2020;382:1708–20. 10.1056/NEJMoa2002032 32109013 PMC7092819

[24] Bi Q, Wu Y, Mei S, Ye C, Zou X, Zhang Z, et al. Epidemiology and transmission of COVID-19 in 391 cases and 1286 of their close contacts in Shenzhen, China: a retrospective cohort study. Lancet Infect Dis. 2020;20:911–9. 10.1016/S1473-3099(20)30287-5 32353347 PMC7185944

[25] O’Driscoll M, Harry C, Donnelly CA, Cori A, Dorigatti I. A comparative analysis of statistical methods to estimate the reproduction number in emerging epidemics with implications for the current COVID-19 pandemic. Clin Infect Dis. 2020;ciaa1599; Epub ahead of print. 10.1093/cid/ciaa1599 33079987 PMC7665402

[26] Baker M, Kvalsvig A, Verrall AJ, Telfar-Barnard L, Wilson N. New Zealand’s elimination strategy for the COVID-19 pandemic and what is required to make it work. N Z Med J. 2020;133:10–4. 32242173

[27] Strøm M, Kristiansen MF, Christiansen DH, Weihe P, Petersen MS. Elimination of COVID-19 in the Faroe Islands: effectiveness of massive testing and intensive case and contact tracing. Lancet Reg Heal–Eur. 2020 Dec 29 [Epub ahead of print]. 10.1016/j.lanepe.2020.100011 PMC7746139 34173620

