# Correction: Vol. 27, No. 3

**DOI:** 10.3201/eid2810.C12810

**Published:** 2022-10

**Keywords:** errata, erratum, correction

The name of author Tróndur Høgnason Mohr was misspelled in Epidemiology and Clinical Course of First Wave Coronavirus Disease Cases, Faroe Islands (M.F. Kristiansen et al.). The article has been corrected online (https://wwwnc.cdc.gov/EID/article/27/3/20-2589_article).

